# Light and Cognition: Roles for Circadian Rhythms, Sleep, and Arousal

**DOI:** 10.3389/fneur.2018.00056

**Published:** 2018-02-09

**Authors:** Angus S. Fisk, Shu K. E. Tam, Laurence A. Brown, Vladyslav V. Vyazovskiy, David M. Bannerman, Stuart N. Peirson

**Affiliations:** ^1^Sleep and Circadian Neuroscience Institute (SCNi), Nuffield Department of Clinical Neurosciences, University of Oxford, Oxford, United Kingdom; ^2^Department of Experimental Psychology, University of Oxford, Oxford, United Kingdom; ^3^Department of Physiology, Anatomy and Genetics, University of Oxford, Oxford, United Kingdom

**Keywords:** learning and memory, alertness, circadian disruption, sleep disruption, melanopsin

## Abstract

Light exerts a wide range of effects on mammalian physiology and behavior. As well as synchronizing circadian rhythms to the external environment, light has been shown to modulate autonomic and neuroendocrine responses as well as regulating sleep and influencing cognitive processes such as attention, arousal, and performance. The last two decades have seen major advances in our understanding of the retinal photoreceptors that mediate these non-image forming responses to light, as well as the neural pathways and molecular mechanisms by which circadian rhythms are generated and entrained to the external light/dark (LD) cycle. By contrast, our understanding of the mechanisms by which lighting influences cognitive processes is more equivocal. The effects of light on different cognitive processes are complex. As well as the direct effects of light on alertness, indirect effects may also occur due to disrupted circadian entrainment. Despite the widespread use of disrupted LD cycles to study the role circadian rhythms on cognition, the different experimental protocols used have subtly different effects on circadian function which are not always comparable. Moreover, these protocols will also disrupt sleep and alter physiological arousal, both of which are known to modulate cognition. Studies have used different assays that are dependent on different cognitive and sensory processes, which may also contribute to their variable findings. Here, we propose that studies addressing the effects of different lighting conditions on cognitive processes must also account for their effects on circadian rhythms, sleep, and arousal if we are to fully understand the physiological basis of these responses.

## Introduction

Light exerts profound effects on physiology and behavior, including entraining circadian rhythms as well as having direct effects on body temperature, melatonin, cortisol, and the cortical electroencephalogram (EEG) (1–4). These effects of light are of particular concern in the modern 24/7 society as inappropriate light exposure affects an increasing proportion of the populace. This includes not only shift work and jet-lag, but exposure to light at night and the effects of light emission from mobile devices, such as laptops, tablets, and smartphones (5). Extended periods of abnormal light exposure can result in circadian disruption, which has been implicated in changes in metabolism, sleep, and cognition as well as increasing the risk of metabolic and cardiovascular disease (6). Many studies of circadian disruption in animal models have involved exposure to abnormal light/dark (LD) cycles (7–10). While these studies have been critical for understanding how circadian disruption affects different systems, the relationship between the direct effects of light and the long-term consequences of abnormal light exposure are not straightforward. Specifically, abnormal LD cycles may affect physiology *via* the direct effects of light as well as *via* its effects on the circadian system. Changes in circadian function may in turn influence sleep, which will subsequently affect additional processes. Here, we provide an overview of the mechanisms mediating photoentrainment before going on to summarize the effects of light on sleep, arousal, and cognitive processes. We then summarize the effects of circadian disruption on cognition in the context of these different mechanisms, with a particular focus on how abnormal light exposure may influence cognitive function.

## Circadian Rhythms

Circadian rhythms are approximately 24 h cycles in physiology and behavior that enable an organism to predict and adapt to periodic changes in its environment. These rhythms provide a selective advantage, enabling anticipation and exploitation of predictable changes (11, 12). Circadian rhythms have been described in virtually all organisms, from cyanobacteria to mammals. Moreover, they have been shown to coordinate numerous aspects of physiology and behavior, influencing everything from locomotor and sleep/wake cycles to hormonal rhythms, metabolism and cognitive performance (13). Conversely, disrupted circadian rhythms impair fitness. Studies on ground squirrels with SCN lesions found that they were predated 20% more than sham-operated control animals (14), and cyanobacteria with differing circadian periods showed increased fitness when their period matches that of the prevailing LD cycle (15). Due to the role of the circadian system in optimizing physiology and behavior in anticipation of predictable environmental changes, a mismatch between internal circadian time and the external LD cycle appears to be a key mechanism by which circadian disruption gives rise to negative health consequences (16). In the following section, the anatomical and molecular basis of circadian rhythms is described, along with the mechanisms by which these rhythms are entrained to the external environment.

### The Suprachiasmatic Nuclei (SCN)

In mammals, the master circadian pacemaker is located within the paired SCN of the anterior hypothalamus. When the SCN are lesioned, animals become arrhythmic (17–19). Furthermore, if fetal SCN are transplanted into an SCN lesioned animal, circadian rhythms are restored (20), with a period determined by the donor animal (21). The SCN show circadian variations in electrical activity and firing rate over 24 h, with high activity during the subjective day and low activity during the subjective night (22). Individual SCN neurons oscillate with a period of roughly 24 h when dissociated from the rest of the SCN tissue, indicating that these rhythms are generated at an intracellular level rather than occurring as an emergent network property (23). While the role of the SCN in driving circadian rhythms in physiology and behavior in mammals was established in the 1970s, it was not until the late 1990s that the molecular basis of these rhythms was established.

### The Molecular Circadian Clock

**Figure 1:**
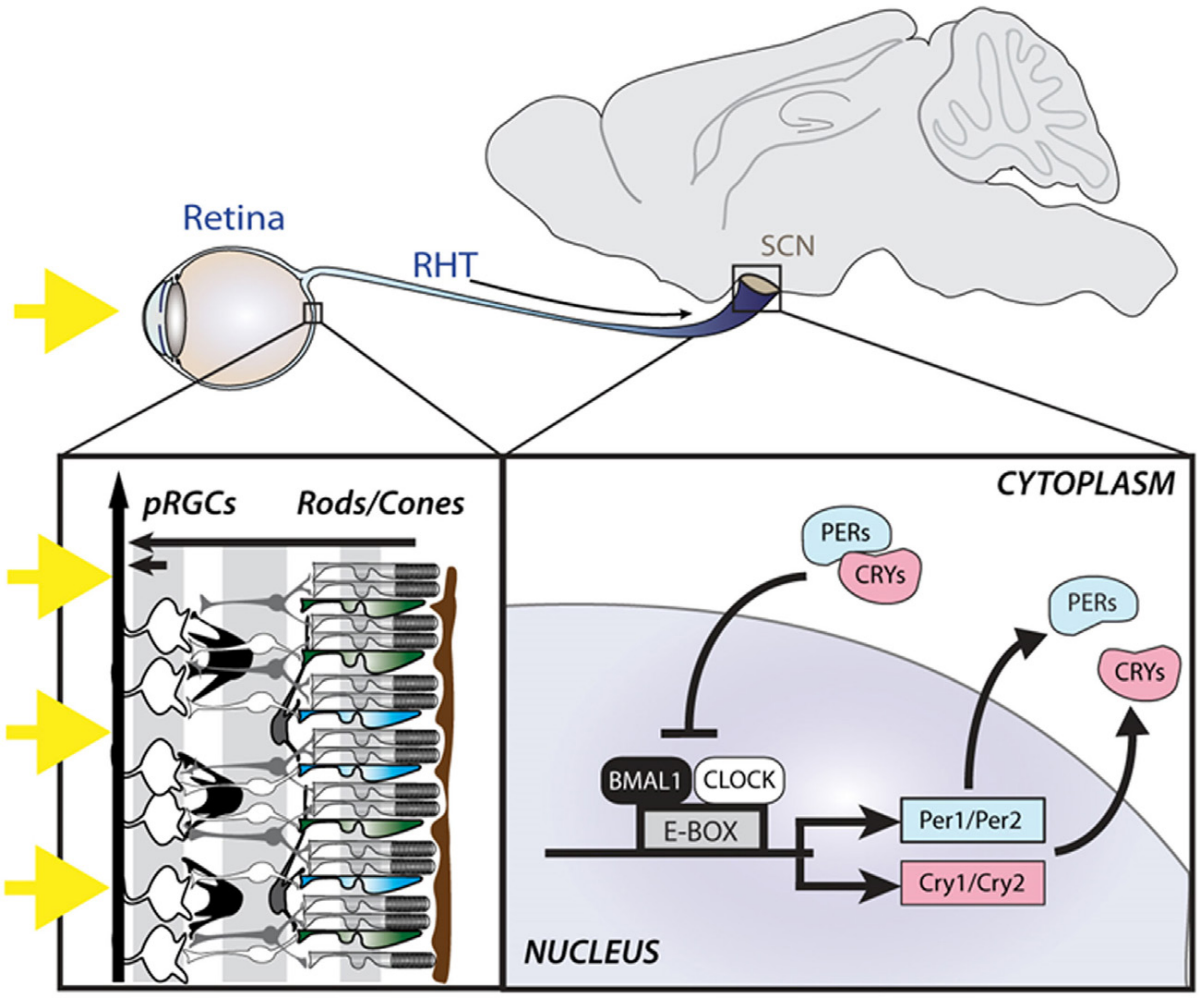
Light is detected by the photoreceptors of the retina, including the rods and cones as well as photosensitive retinal ganglion cells (pRGCs), expressing the photopigment melanopsin. Light information is relayed to the suprachiasmatic nuclei (SCN) *via* the retinohypothalamic tract (RHT), where it entrains an intracellular molecular clock mechanism, consisting of positive (BMAL1 and CLOCK) and negative (PER and CRY) elements.

The underlying mechanism generating intracellular circadian rhythms is a transcriptional-translational feedback loop (TTFL) comprising positive, negative, and accessory limbs (24). The positive limb consists of the core clock proteins, CLOCK and BMAL1, which both contain a basic helix-loop-helix domain and bind together to form heterodimers. These in turn bind to E-box enhancer regions of *Per1-2* and *Cry1-2* genes to promote their transcription (Figure 1). The negative limb comprises the translated PER and CRY proteins which translocate back into the nucleus and directly interact with the CLOCK/BMAL1 complex to inhibit transcription. In turn, the transcription of PER and CRY proteins are reduced, and the proteins are also actively broken down, leading to re-activation of transcription by CLOCK/BMAL1. In addition to the core loop, an accessory loop is also driven by the CLOCK/BMAL1 activation. The *Rev-erb*α gene is transcribed and produces the orphan nuclear receptor REV-ERBα, which activates a ROR response element in the promoter of *Bmal1* to inhibit its transcription. As PER and CRY interact with CLOCK/BMAL1 to inhibit transcription, *Rev-erb*α falls as well, disinhibiting *Bmal1* transcription, allowing levels to rise again (25). CLOCK/BMAL1 heterodimers also drive the transcription of a large number of other genes which contain E-box enhancers in their promoter region, termed clock controlled genes (CCGs), thus allowing the clock to influence a wide range of cellular functions (26). The characterization of the molecular basis of circadian rhythms reflects one of the best examples of how genetic mechanisms can give rise to complex behavior. Indeed, it is remarkable that changes in a single gene can give rise to changes in the period of the circadian clock, or even arrhythmicity.

### Light Input to the Circadian Clock

A clock is of no use unless it can be set to the right time. In mammals, the master SCN clock is entrained to the external environment by time cues (zeitgebers), the most important of which is light detected by the eyes. Indeed, loss of the eye abolishes entrainment (27, 28). The SCN receives input from the retina *via* the retinohypothalamic tract (RHT), which allows the light to adjust the phase of its endogenous rhythms to match that of the environment (29). Studies on the photoreceptors mediating circadian entrainment demonstrated that mice can still phase shift their locomotor activity rhythms and suppress pineal melatonin in response to light even in the absence of the classical rod and cone photoreceptors that mediate vision (30, 31). These findings suggested the existence of a novel retinal photoreceptor, in addition to the well-characterized rods and cones. It was subsequently shown that a subset of photosensitive retinal ganglion cells (pRGCs) expressing the photopigment melanopsin (OPN4) are intrinsically light sensitive and form the primary projection to the SCN (Figure 1) (32). However, as pRGCs receive input from the outer retina, their output *via* the RHT depends upon both their intrinsic responses as well as extrinsic signals from rods and cones (33, 34). As such, mice lacking melanopsin (*Opn4^−/−^*) display normal circadian entrainment, but attenuated phase shifting responses to light (35, 36). However, mice in which pRGCs are genetically lesioned are unable to entrain to light (37). Since the identification of pRGCs, it has become clear that these cells mediate more than just circadian entrainment, and are involved in a range of non-image forming (NIF) responses to light, including the pupillary light response, regulation of sleep–wake timing, photophobia, light aversion, and cognitive function, as well as influencing image forming responses, such as visual adaption (38). Melanopsin-expressing pRGCs project to multiple brain targets, including the intergeniculate leaflet, olivary pretectal nucleus, medial amygdala, lateral habenula, and superior colliculus, suggesting that different NIF responses may involve different neural projections (38, 39).

The primary neurotransmitters of the RHT are glutamate and pituitary adenylate cyclase activating polypeptide, which are released at synapses in the SCN in response to photic stimuli (40, 41). This results in increases in calcium concentration and firing rates in SCN neurons. Increased calcium concentration activates intracellular signaling pathways [e.g., cyclic AMP (cAMP) and PKA], converging on the phosphorylation of cAMP response element binding protein, which translocates to the nucleus, binding to cAMP response elements in the promoters of *Per1* and *Per2*, increasing their transcription. This results in the molecular clock in SCN being either advanced or delayed. Although this link between light input, membrane events, and the TTFL has been characterized, the mechanisms by which the TTFL regulates membrane potential are poorly understood (3, 42). While great progress has been made in understanding the molecular basis of photoentrainment, this model is almost certain to be incomplete.

### Clock Outputs and Peripheral Clocks

The SCN has widespread projections throughout the brain, including to the septum, the anterior paraventricular thalamus, and to multiple hypothalamic nuclei including the subparaventricular zone, ventromedial hypothalamus, dorsomedial hypothalamus, and pre-optic area (43). The SCN also projects to the paraventricular nucleus, whereby it modulates circadian rhythms in neuroendocrine and autonomic function (44, 45). Furthermore, molecular circadian rhythms are not confined to the SCN. Studies in the late 1990s showed that rat fibroblasts, which had been cultured for 30 years, were capable of rhythmic clock gene expression following a serum shock (46). Furthermore, clock gene reporter studies demonstrated that tissues throughout the body displayed rhythmic clock gene expression, including the liver and adrenal glands (47). These peripheral clocks are thought to play a key role in regulating local tissue physiology (48), with the SCN coordinating circadian timing throughout the body *via* a combination of neural, paracrine, hormonal, and behavioral signals. The identification of peripheral clocks throughout the body led to a fundamental change in our understanding of circadian rhythms, demonstrating that temporal organization is embedded in the physiology of virtually all cells, tissues, and organs.

## Sleep

The sleep/wake cycle is perhaps the most familiar consequence of our circadian rhythms. While sleep is modulated by the circadian system, it is also critically regulated by a homeostatic drive that increases with extended waking. As such, sleep and wakefulness depend upon the interaction between these circadian and homeostatic processes (49, 50). Sleep is a complex process involving multiple brain regions and a network of mutually inhibiting arousal and sleep-promoting neurons (51–53). This involves wake active nuclei in the brainstem, hypothalamus, and the basal forebrain that fire during waking, and become less active during both NREM and REM sleep.

Sleep can be defined in both behavioral and physiological terms. Behaviorally, it involves a period of extended inactivity, with increased arousal threshold, a species-specific body posture and a typical sleep site (54). Physiologically, sleep is defined by the EEG, which measures electrical activity at the level of the cortex. During sleep, well-characterized changes occur in the EEG, classified as rapid-eye movement (REM) sleep and non-rapid-eye movement (NREM) sleep (55). NREM sleep involves synchronized rhythmic EEG activity, occurring widely over the cortex, reflecting changes in the firing pattern of cortical neurons. By contrast, REM sleep is characterized by an EEG similar to the awake state, but with atonia and REMs (56). The brain cycles through these stages several times through the night in humans (in rodents, many more such cycles occur), with higher levels of NREM sleep at the start of the night, and higher REM sleep occurring later. While the precise function of sleep is not fully understood, it is likely it subserves multiple functions, including metabolite clearance, memory processing, immune restoration, and other functions (57). Deprivation of sleep has many negative consequences, including cognitive impairment (58), metabolic dysregulation (59), and following extended sleep deprivation, eventually death (60).

### Homeostatic and Circadian Regulation of Sleep

**Figure 2:**
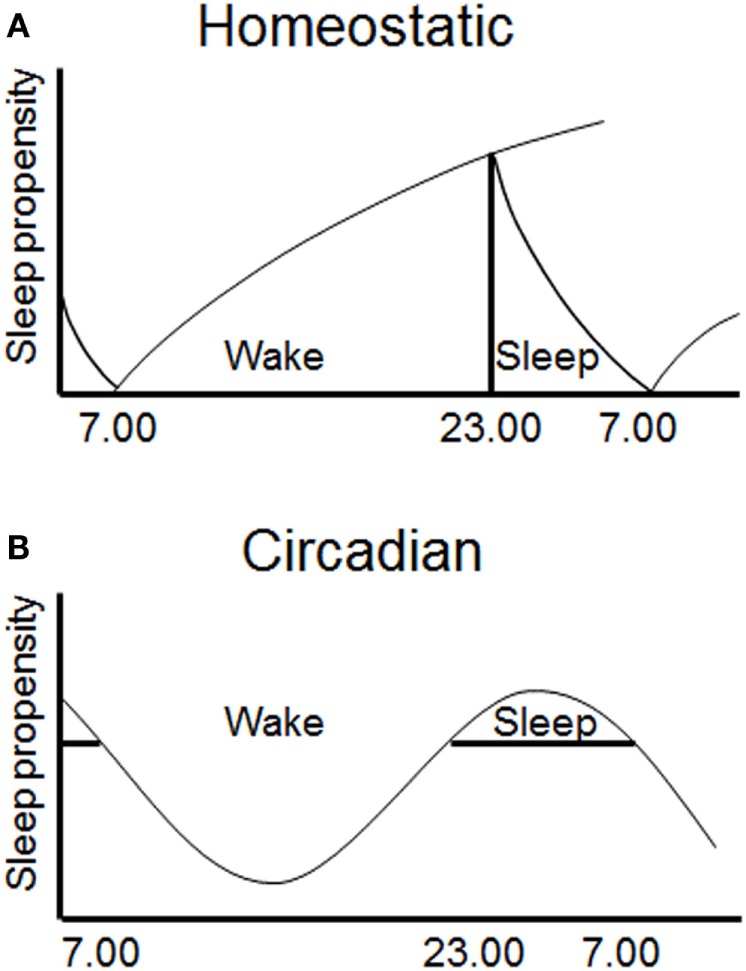
Sleep is regulated by two interacting processes. **(A)** A homeostatic mechanism (Process S) increases the requirement for sleep with prolonged waking and dissipates during sleep. **(B)** A circadian mechanism (Process C) provides a drive for wakefulness at specific phases of the 24 h cycle (49).

The quantity, quality, and timing of sleep and wakefulness are regulated by both a homeostatic and a circadian process (termed Process S and C, respectively) (50). These processes interact to produce periods of wake and sleep during the day (Figure 2). This conceptual model has been useful in interpreting disturbances of sleep/wake regulation, and has been validated by quantitative predictions (61–63). The homeostatic process gradually accumulates during prior wakefulness, and dissipates during sleep. This process is highly correlated with the power of slow wave activity (SWA) on the EEG during NREM sleep, characterized by frequencies in the 0.5–4 Hz range (64). The mechanisms underlying this homeostatic process are unclear. However, a number of putative sleep factors build up in the brain during prolonged wakefulness and dissipate during sleep and these may mediate the homeostatic process (65). Perhaps the best known example is adenosine, which increases in the basal forebrain during wake and dissipates during sleep, and has been proposed to account for the action of adenosinergic drugs, such as caffeine, on sleep (66, 67).

The circadian process varies throughout the 24 h day, allowing wake and sleep at alternate phases of the cycle (68). The primary driver of the circadian process is thought to be the SCN, as following SCN lesions endogenous rhythms in rest/activity are abolished, but the homeostatic regulation of sleep remains intact (69–71). The SCN appears to primarily provide a wake-promoting signal during the active period as lesions increase the total amount of sleep time (72). Studies in which internal desynchrony is induced in rats using 22 h days have also shown that rhythms in NREM sleep can be dissociated from rhythms in body temperature and REM sleep (73). More specifically, the circadian timing of REM sleep has been shown to be associated with clock gene expression in the dorsomedial SCN (74). The SCN likely mediates process C *via* direct and indirect projections to hypothalamic and brainstem nuclei (including locus coeruleus, VLPO, and orexin neurons), which control levels of arousal and sleep (75, 76). The ventral subparaventricular zone appears to be a key node in this pathway, as lesions of this nucleus result in profound reduction in rhythms of locomotor activity and sleep (77). Together, the homeostatic and circadian processes determine the timing of sleep and wake, and disturbance of either process has the potential to disrupt sleep and influence subsequent waking performance.

### Direct Effects of Light on Sleep

In addition to the role of homeostatic and circadian processes in the regulation of sleep, light exposure also directly modulates sleep. While light increases arousal and alertness in diurnal species such as humans, it promotes sleep in nocturnal rodents. Studies in rats have shown that light exposure suppresses activity and results in increased sleep, whereas darkness results in increased wakefulness (49, 78). One of the many projections of melanopsin pRGCs is to the sleep-promoting VLPO (39). This observation, coupled with the role of melanopsin pRGCs in the regulation of numerous NIF responses to light, led to studies of acute sleep induction in mice lacking melanopsin (*Opn4^−/−^*). Initial studies suggested that melanopsin-deficient mice show impaired sleep induction in response to nocturnal light exposure, suggesting that melanopsin plays a key role in mediating sleep induction and maintenance in response to light (79–82). However, these findings were not consistent with data showing that nocturnal light exposure in rodents produces a rise in plasma corticosterone (83), and that these effects on adrenal corticosterone are *via* the SCN, but independent of effects on the clock (84). Recent studies using different wavelengths of light have shown that short-wavelength 470 nm (blue) light results in delayed sleep onset, coupled with behavioral light aversion and elevated plasma corticosterone, and that this arousal response is attenuated in melanopsin-deficient mice. By contrast, longer wavelength 530 nm (green) light of the same intensity resulted in reduced arousal responses and more rapid sleep induction. Consistent with previous studies, sleep induction in response to green light was attenuated in melanopsin-deficient mice (85). These data are consistent with recent data using chemogenetic activation of pRGCs that produce behavioral arousal, rather than sleep (86). Furthermore, these findings are also consistent with the alerting effects of light described in humans (see Direct Effects of Light on Cognitive Processes).

The finding that melanopsin may be involved in the regulation of the sympathetic nervous system and subsequent arousal is perhaps not surprising, as pRGCs provide a major input to the SCN which is known to regulate sympathetic function. Indeed, this is the primary pathway *via* which pineal melatonin synthesis is regulated (87). By contrast, explaining why melanopsin-deficient animals show impaired sleep induction in response to light is more challenging. One potential explanation is that melanopsin has recently been shown to be involved in light adaptation (88). If rod/cone signaling normally mediates sleep induction, in the absence of melanopsin responses to bright light stimuli may quickly saturate leading to impaired responses compared to wild-type mice (85). Rather than impaired photic input, an alternative explanation is that the impaired sleep induction in melanopsin-deficient mice may simply reflect a reduced requirement for sleep. Support for this hypothesis comes from data from melanopsin knockout mice showing reduced delta power during the dark phase as well as reduced accumulation of delta power following sleep deprivation. These findings suggest that the need for sleep increases at a slower rate in melanopsin-deficient mice (82). If this is indeed the case, it would suggest that differences in homeostatic sleep could account for impaired sleep induction in melanopsin-deficient mice, rather than deficits in light input to the VLPO as has previously been suggested.

Additional experimental variables may influence sleep induction in response to light. The environmental context is almost certain to influence acute sleep induction, as light exposure in the home cage may produce quite different effects on sleep in comparison to a novel environment. For example, in novel environments, such as an open field or novel object testing arena, sleep induction is not observed in response to light (89, 90). In addition, it should also be considered that while c-Fos has been used as a marker of VLPO activation during sleep (91), induction of Fos in response to light may simply reflect the subsequent sleep/wake status of the animal rather than providing a marker of light input as has been widely used in the SCN (92). While there is a limited retinohypothalamic projection from melanopsin pRGCs to the VLPO (39, 93, 94), it is quite possible that the direct effects of light on sleep may be mediated *via* other neural pathways.

In summary, as well as circadian and homeostatic processes, light can also directly modulate sleep. However, future studies are required to understand how circadian and homeostatic processes interact to influence acute sleep induction in response to light, as well as the detailed neural pathways involved. These direct effects of light on sleep, and conversely alertness, are clearly important for the effects of light on cognitive processes.

## Cognition

Given the widespread influence of the circadian system across multiple aspects of physiology and behavior, it is not surprising that cognitive processes also display circadian rhythms. While learning and memory provide easily testable and translatable paradigms in both human and animal models, several other processes such as attention, mood, and reaction time also show circadian variation (95). As such, the influence of light and circadian rhythms on cognition are unlikely to be due to effects on a single process.

### Circadian Regulation of Cognitive Processes

In humans, cognitive function shows variation over the 24 h day, starting off low in the morning, maintaining high levels until habitual bedtime, apart from a dip in the afternoon. This pattern is related to both sleep and circadian processes. Forced desynchrony, in which subjects are exposed to a light schedule to which they are unable to entrain, have been used to investigate the role of circadian and homeostatic regulation of cognitive processes, such as alertness, vigilance, working memory, sleepiness, and mood. These studies show circadian rhythms in all of these different cognitive processes, which also decline with time awake (96, 97).

Again, data from animal studies are less conclusive. Some studies report increased performance during the subjective night in aversive (98) and appetitive tasks (99, 100), whereas others found better performance during the subjective day (89, 101, 102). These contradictory findings may reflect the nature of the behavioral tasks employed. Originally, it was shown that rodents performed best on behavioral tasks when training and testing times were matched, which may reflect state/context dependent learning (103, 104). To date, a major limitation of circadian studies of different cognitive processes is that while circadian time is carefully controlled, the preceding sleep/wake status of the animal is rarely considered.

In summary, processes such as learning and memory certainly appear to be under circadian control to some extent, and this circadian regulation has the potential to influence the effects of light on cognition.

### Direct Effects of Light on Cognitive Processes

Human studies have demonstrated an important role of light in the regulation of alertness. Imaging studies have also shown that light exposure can influence cortical and subcortical networks involved in cognitive processes, such as attention, arousal, and memory (105–110). In addition, a number of studies have shown that short-wavelength light (470 nm or lower) is associated with the increased suppression of melatonin, reduction in subjective sleepiness, reduced reaction times and changes in EEG power in the delta–theta frequency range (111, 112). These findings are consistent with recent studies on the effects of light-emitting devices on subjective alertness, EEG and sleep latency (113). The primary cognitive effects of light on appear to be *via* increased alertness—which is typically measured using subjective rating scales as well as tests of sustained attention such as the psychomotor vigilance task, a simple reaction time task. Overall, these studies suggest an increase in subjective ratings of alertness in response to light, though whether these findings always translate into increased cognitive performance is less clear (114–116). However, in cognitive tasks where sustained attention is necessary, light may be expected to exert greater effects.

Despite the wealth of human studies on the acute effects of light on alertness, remarkably few animal studies have investigated the effects of light on cognitive performance. Studies on the acoustic startle response in rats have shown that this response is enhanced by increasing light exposure (117). Subsequent studies investigated the effects of light on tone-cued fear conditioning, finding that light enhances freezing responses in wild-type mice (118). Short pulses of white light at night in mice have been shown to improve consolidation of contextual fear learning and long-term potentiation (119). Under other conditions, bright light exposure impaired spatial navigation performance on a water maze task in BALB/c mice, which was associated with increased anxiety and elevated corticosterone levels (120). Most recently, studies on spontaneous object recognition show that bright light during the test phase impairs recognition performance, regardless of the light level during the sample phase [although see Ref. (121)]. These effects on recognition memory are abolished in both mice lacking rods/cones as well as in mice lacking melanopsin, suggesting that integrated responses from both systems mediate the effects of light on performance (90, 118). Light may also influence emotional processes such as mood, and recent studies using aberrant LD cycles have shown that these may give rise to depression-like behaviors (122) (see T-Cycles). These data add a further complexity to the effects of light, which may involve circadian and non-circadian effects (9, 123).

In summary, data from humans show clear effects of light on alertness, and in some cases, also on performance in tests of sustained attention. However, despite a number of rodent studies exploring the effects of light on different cognitive processes, no consistent effects have emerged. Light may certainly affect the outcome of laboratory tests of learning and memory in rodents. However, the direction and amplitude of any effect may depend on the nature of the test and the different cognitive processes involved. The difficulty of characterizing mechanisms in human studies, combined with the lack of consistent effects in animal models, has led to a lack of any detailed understanding of the photoreceptor contributions and underlying neural pathways involved in such responses.

### Sleep and Cognitive Processes

Given the key role of the circadian system in the regulation of sleep, any disruption of the circadian system is likely to influence subsequent sleep/wake timing. Sleep has been suggested to play a role in cognitive performance (124), and sleep disruption is known to impair multiple aspects of cognition, including arousal, attention, and working memory (125). As such, the effects of sleep disruption must also be taken into account when considering the effects of light on cognitive processes, particularly where circadian function is affected.

In rodents, sleep deprivation influences several aspects of memory, which have been assessed using a variety of behavioral tests. Total sleep deprivation in the first 5 h after training has been reported to reduce contextual fear memory, despite otherwise adequate sleep (126–129), but produce no effect on tone-cued fear memories (126). Using the platform-over-water REM sleep deprivation method, contextual fear memory has been suggested to be impaired, again with no effect on tone-cued fear memories (130–133).

Spontaneous object recognition is a highly tractable test of learning and memory in rodents (134, 135) that has also been widely used to study the role of sleep in learning and memory. Studies have shown that both object-recognition and object-location memories are impaired by 5–6 h of sleep deprivation after training (136–139), with an apparently crucial window at 3–4 h (140). Another test that has been used to study the effects of sleep deprivation is the Morris watermaze, which relies on aversive immersion in water to motivate animals to find a hidden platform. While this requires spatial learning and is sensitive to hippocampal damage, other brain regions and strategies are also important (141). 4 h of selective REM sleep deprivation immediately following training has been suggested to impair performance (142). However, this finding is equivocal with some studies agreeing (143–145) and others disagreeing with the results (146). This may be due to differences between protocols favoring different search strategies and/or brain areas. Spontaneous spatial recognition in the Y-maze has also been studied following sleep deprivation. Similar to other behavioral paradigms, performance is impaired by 12 h total sleep deprivation prior to training (147).

In addition to sleep duration, sleep architecture is also important for memory. Humans sleep in a consolidated bout once a day and progress through many cycles of REM and NREM sleep during the night. If this is fragmented, daytime function is impaired and increased sleepiness occurs (148). By contrast, rodents sleep in multiple short bouts, consisting of both REM and NREM sleep, throughout the day and night, with a greater amount of sleep during the light phase, primarily due to increased length of sleep bouts during the day (149). Disturbing sleep architecture with regular waking prevents normal completion of sleep bouts, resulting in increased sleep pressure despite no change to the total sleep duration (150). Sleep fragmentation also impacts cognitive processes. Mice subjected to sleep fragmentation for 15 days, induced by being disturbed by a bar across the cage every 2 min, have poor learning and retention in the Morris watermaze (151). Similarly, optogenetic activation of hypocretin neurons fragments sleep without altering total sleep time, and when fragmented in the first 4 h following training, causes deficits in object-recognition memory (152). Importantly these studies suggest that even when sleep timing and total sleep duration may remain comparable, fragmented sleep can give rise to impaired performance in specific cognitive processes.

Together the data described above suggest that sleep disruption impairs specific aspects of learning and memory. However, these effects are not straightforward, with sleep deprivation, selective REM deprivation and sleep fragmentation having subtly different effects on different behavioral tests. Some of the cognitive deficits that occur as a result of sleep loss may arise as a result in specific changes in synaptic function, particularly relating to glutamatergic signaling and synaptic plasticity. One example of this is the role of the GluA1 AMPA receptor subunit, encoded by the *Gria1* gene. The GluA1 subunit is important in both AMPA receptor trafficking and synaptic plasticity (153–155). Critically, GluA1 levels in synaptoneurosomes in both the cortex and hippocampus have been shown to be elevated following prolonged wakefulness (156). This supports the synaptic homeostasis hypothesis, whereby wakefulness is associated with a net increase in synaptic strength, which is subsequently renormalized during sleep (157). Data from mice lacking GluA1 may provide some insight into the consequences of these changes in synaptic plasticity that occurs during sleep. GluA1-deficient mice show unimpaired performance on associative, long-term memory tasks, such as the Morris watermaze. By contrast, these animals show selective deficits in short-term habituation to recently experienced stimuli (158, 159). These findings suggest that during waking synaptic GluA1 levels increase, reflecting an ongoing habituation and reduction in attention. This hypothesis suggests that sleep may be important for the restoration of attentional performance (160).

In conclusion, while the disruption of sleep undoubtedly influences cognitive function, the specific cognitive processes affected and the underlying mechanisms involved are not straightforward. However, when considering the effects of light—either directly or *via* its effects on the circadian system—researchers should always be aware that effects on cognition could arise due to a concomitant disruption of sleep.

### Effects of Arousal on Cognitive Processes

Arousal is another key factor that may influence the outcome of studies on cognition. In this context, rather than simply being awake, arousal refers to a state of physiological alertness resulting in increased attention and cortical activity, along with changes in motivation and emotional state. Again, light may modulate arousal either directly, or indirectly *via* the circadian modulation of arousal. Arousal responses are thought to be mediated *via* projections from the SCN to the dorsomedial hypothalamus which are then relayed to the ascending arousal system, including the noradrenergic locus coeruleus, cholinergic laterodorsal tegmental nuclei, dopaminergic ventral tegmental area and serotoninergic raphe nuclei (75). In addition, the widespread projections of the melanopsin pRGCs may also be important in the effects of light on arousal, including the lateral habenula, medial amygdala, and subparaventricular zone (9, 39). In addition to the regulation of the ascending arousal system, physiological arousal may also be accompanied by increased activity of the sympathetic branch of the autonomic nervous system, resulting in widespread changes in physiology, particularly relating to cardiovascular and adrenal function. Whether these effects of light on arousal are independent or interrelated remains unclear.

**Figure 3:**
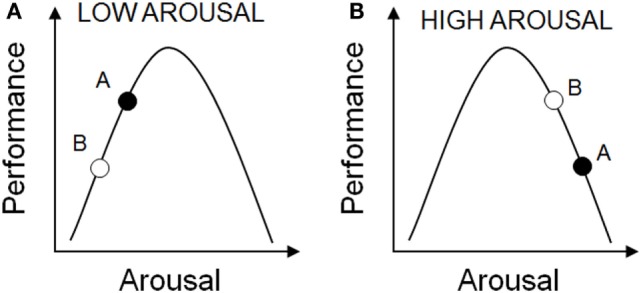
The relationship between arousal and cognitive performance. The effects of circadian time on performance may depend upon different levels of arousal. These may in turn be integrated with other arousal-promoting stimuli. **(A)** Under normal low arousal conditions, performance may be better at circadian time A versus circadian time B. **(B)** However, under conditions of high arousal, these circadian time-dependent effects may result in better performance at time B versus time A, due to excessive arousal.

The relationship between arousal and cognitive performance is complicated. Yerkes and Dodson (161) reported that with simple learning tasks there was a positive linear relationship between arousal and performance (i.e., the higher the level of arousal, the better the task performance). However, as the difficult of the task increased, an inverted-U shaped relationship between arousal and cognitive performance was observed, with optimal performance requiring an optimal level of arousal (161). As such, rhythms in arousal will influence where an individual sits on the arousal-performance curve, and are likely to contribute to the rhythms seen in behavioral performance in animal studies. As a result of the differences in baseline arousal, the response to stressors such as handling, restraint, and environmental noise will differ (162, 163). When baseline arousal is low, increased arousal may be expected to result in improved performance, but when baseline arousal is high, increasing arousal further may impair performance (Figure 3).

The circadian control of adrenal glucocorticoids via the classical hypothalamic–pituitary–adrenal (HPA) axis is well known (164, 165). As well as the circadian regulation of adrenal glucocorticoids, light has also been shown to directly modulate glucocorticoid release (83), and may also exert different effects on arousal depending upon wavelength (85). This acute response to light does not involve the classic HPA axis, instead relying upon modulation of the sympathetic nervous system (83). While transient increases in glucocorticoids in response to stressors—such as light exposure—are a normal physiological response (termed “allostasis”), long-term exposure to such stimuli can result in fundamentally different responses (“negative allostasis”) (166). Such chronic stress increases baseline glucocorticoid levels and attenuates the amplitude of glucocorticoid rhythms, both at an ultradian and circadian level (164, 167). Elevated glucocorticoid levels are known to affect cognitive processes, such as learning and memory (168). As well as changes in plasma glucocorticoid levels, cardiovascular markers such as heart rate may provide useful markers of arousal. Furthermore, it has recently been suggested that spontaneous fluctuations in locomotor activity may provide a useful marker of generalized arousal (169, 170), which may be beneficial for future studies in this field.

Given the role of the circadian clock in regulating the ascending arousal system as well as the autonomic nervous system, as well as the direct effects of light on these systems, changes in arousal state should also be considered when investigating the effects of light on cognitive processes.

## Circadian Disruption

The circadian system has evolved to enable organisms to anticipate and exploit predictable changes in the external environment, optimizing physiology and behavior to specific times of day. However, our modern 24/7 society produces numerous examples where lifestyle is in conflict with our internal biological clocks, including shift work and jet-lag. Moreover, artificial light results in light exposure at inappropriate times of day, including light at night as well as exposure to light from mobile devices, such as phones, tablet, and computers. As a result, there is growing concern regarding the consequences of circadian disruption and aberrant light exposure on human health, including effects on metabolism, cardiovascular function, mental health, and even cancer risk (6, 16, 171). Those routinely exposed to such conditions may develop problems with poor performance, insomnia, emotional disturbances, and gastrointestinal complaints. Such symptoms are thought to affect 5–10% of those involved in shift work. Treatments seek to realign the internal clock with the external environment, using scheduled light exposure (or avoidance), short naps, or use of pharmacological interventions, such as melatonin, caffeine, or even prescription drugs (172, 173). With our increasing exposure to artificial light sources, such problems look likely to rise in the future.

To investigate the mechanisms underlying the adverse health outcomes of circadian disruption, an increasing number of studies have investigated the effects of aberrant light exposure on cognitive function, using rodents housed under abnormal LD cycles. However, such abnormal LD cycles may exert their effects *via* different mechanisms. Some result in light exposure during the normal subjective night, whereas others result in a mismatch between internal circadian time and external environmental time, requiring a constant phase adjustment. It has been suggested that this mismatch may be the basis of the negative consequences of circadian disruption (16). Evidence for this comes from studies in which animals show impaired health under non-24 h environmental conditions (16). Perhaps the best evidence comes from studies on longevity, where wild-type mice show reduced lifespan under non-24 h LD cycles (174), and tau mutant hamsters show impaired longevity under 24 h conditions, but normal longevity in constant darkness (175, 176). These studies indicate that a circadian clock is only beneficial if its period matches to that of the environment. An alternative hypothesis for the adverse effects of circadian disruption is that such conditions result in internal desynchrony—where circadian clocks in different tissues (or even different brain regions) may become misaligned or even arrhythmic, resulting in impaired performance (96). Internal desynchrony has been described as a result of scheduled feeding in mice, resulting in desynchrony between clocks in the SCN and hippocampus and impaired learning and memory [(177); eLife], as well as within neuronal subpopulations of the SCN in rats housed under 22 h T cycles, resulting in depression-like behavior (178). The mismatch between internal and external time and internal desynchrony are not mutually exclusive hypotheses, and may both contribute to the negative effects of circadian disruption.

Abnormal LD cycle protocols used to study circadian disruption include constant light (LL), jet-lag, T cycles, dim light at night and disruptive phase shifts (DPSs). The effects of these protocols are summarized in Table 1 and described in detail below with regard to their known effects on circadian physiology, sleep, arousal, and cognitive processes.

**Table 1:** Effects of different abnormal light–dark cycles on circadian rhythm, sleep, arousal, and performance.

Conditions	Circadian	Sleep	Arousal	Cognitive
Constant light	↑ Internal period length (nocturnal)	?	↑ Or ↓ glucocorticoid (e.g., CORT) levels	↓ Spatial performance
	↑ mPER2 expression in suprachiasmatic nuclei (SCN)			↓ Contextual fear conditioning
	↓ SCN neuronal firing			↓ Passive avoidance
	↓ Amplitude in peripheral tissues			↓ Appetitive response timing
	Behavioral arrhythmia			

Jet lag	↓ Locomotor/exploratory activity	↓ Total sleep	↑ CORT response to aversive stimuli	↓ Spatial performance
	Alter phase relationships between SCN and peripheral tissues	↑ Rapid-eye movement (REM) sleep		↓ Appetitive response timing
		Fragmented sleep		↓ Conditioned place preference

Non-24 h T-cycles^a^	↑ Internal period length (nocturnal)	Desynchronize core body temperature and REM sleep	↑ CORT level	↓ Passive avoidance
		↑ Slow-wave activity (sleep)		↓ Spatial performance
		Alter θ and γ power (wake)		↓ Object-recognition performance

Dim light at night	↓ Locomotor/exploratory activity	↓ Amplitude in REM and non-rapid-eye movement rhythms	↓ CORT rhythm	↓ Spatial performance
	↓ Amplitude of activity rhythm			↑ Anxiety-related behavior
	↓ Amplitude of mPER1/2 rhythms			↑ Depression-related behavior

Disruptive Phase Shift^b^	↓ Clock gene expression in SCN	↑ Daytime sleep	?	↓ Object-recognition performance
	Arrhythmia (activity, core body temperature, melatonin)			↓ Spatial alternation performance

*^a^T7, T20, or T22 cycles*.

*^b^Hamsters only*.

*↑ = Increase, ↓ = Decrease; ? = No published studies available. See text for details and references*.

### Constant Light

Constant conditions are frequently used in circadian research to study free-running circadian rhythms. While constant darkness allows animals to organize their behavior exclusively according to their internal circadian clock, constant light has been used to study light input as well as a means of producing circadian disruption.

#### Circadian

Constant light results in an intensity-dependent lengthening of the period of nocturnal animals, and can cause complete arrhythmia (179, 180). Both the molecular and electrophysiological timekeeping of the SCN is altered. Long-term constant light exposure leads to constitutively higher levels of mPER2 (181), and clock gene rhythms become gradually desynchronized (182). At an electrophysiological level, as well as period lengthening, the amplitude of SCN firing is reduced and firing rate is more variable (183). Peripheral clocks have also been shown to be affected, resulting in dampened amplitude and broadened peak phases (184), comparable with SCN lesions (185).

#### Sleep

Since constant light alters circadian activity, it will also affect sleep distribution. However, we are aware of no detailed characterization of the effects of constant light on the amount, distribution, and architecture of sleep.

#### Arousal

Constant light may influence the HPA axis and alter circulating glucocorticoid levels. However, the results of these studies are equivocal, with some studies finding reduced plasma corticosterone (183, 186, 187), some finding increased levels (188–190) and others finding no effect (191). One potential explanation for these conflicting results is the ultradian pulsatility in glucocorticoid secretion. This plays a key role in glucocorticoid signaling, but is only detectable using high-resolution sampling (192).

#### Cognitive Effects

Constant light has been suggested to impair spatial memory in the Morris watermaze, as well as in contextual fear memory and passive avoidance (193–197). However, longer durations (5–7 weeks) of constant light produce no change in a plus-maze discriminative avoidance task (198). Interval timing has also been reported to be disrupted by constant light (199). Recent studies using repeated constant light on different aspects of recognition memory have shown a dampening of SCN clock gene rhythms, resulting in desynchrony between clocks in the SCN, hippocampus, and olfactory bulb (89). As described above, constant light may cause period lengthening and in some cases arrhythmia. However, to date, no studies have related the effects of constant light on cognition to these different circadian effects.

In summary, constant light leads to a lengthening of circadian period or even arrhythmicity, with potential effects on the coupling of central and peripheral clocks. Such conditions have been shown to influence arousal and cognitive processes, though these effects are not consistent between studies.

### Jet Lag

Shifting the LD cycle under which animals are housed has been used to mimic the sudden shift in time-zones produced by jet-lag. Acute jet-lag protocols typically involve a single advance or delay in the LD cycle. In addition, chronic jet-lag—involving repeated shifts of the LD cycle—has also been used as a model of circadian disruption.

#### Circadian

In response to acute jet-lag, rodents typically shift their activity over several days to re-entrain to the new LD cycle. Usually this involves an advance of the LD cycle so that activity onset can be easily determined. Delaying the LD cycle results in suppression of activity by light (negative masking), making the activity onset more difficult to determine. In response to a 6 h advance of the LD cycle, mice shift their activity by ~1 h per day, typically taking 5–6 days to re-entrain. However, patterns of gene expression in the SCN may change more rapidly (200). Peripheral clocks shift at different rates, potentially leading to a differing phase relationship with the SCN while they re-align (200, 201). Chronic jet-lag protocols result in the animal having to repeatedly re-entrain to the shifting environment LD cycle, and may alter the relationship between the SCN and other peripheral circadian oscillators.

#### Sleep

Acute jet-lag has been reported to result in no change in total sleep time, but mild changes to the distribution of sleep (202). However, under chronic jet-lag, sleep is both fragmented and reduced by ~10% per week compared with baseline conditions. While no increase in SWA was reported following chronic jet-lag, an increase in REM sleep and brief arousals was described (203, 204).

#### Arousal

The stress axis is markedly affected by acute jet-lag, enhancing the magnitude of the stress-evoked corticosterone response (202). With chronic jet-lag, baseline corticosterone as well as anxiety and depression-like behaviors have been reported to be unaffected (204).

#### Cognitive

Acute jet-lag impairs spatial memory, whether performed before or after initial training (10, 194, 202, 205, 206), with deficits persisting after re-entrainment (202, 206). Interval timing has been reported to be less accurate after the shift but returns to normal with full behavioral re-entrainment (199). Other tasks, such as a sustained attention task (100) and social memory (207), are unaffected by acute jetlag. Several behavioral tasks show impairment after chronic jet-lag, including conditioned place preference (208, 209), a 8-arm radial arm task (210), and the Morris watermaze (210, 211). Access to a running wheel has been suggested to mitigate some of these effects (210). Finally, fear memory to both tone and context are unimpaired after chronic jet-lag (202, 211).

Together, these data suggest that circadian disruption induced using both acute and chronic jet-lag protocols result in specific changes in cognitive processes. However, given the influence of these protocols on both sleep fragmentation as well as arousal, it is difficult to ascertain the mechanisms by which cognitive processes are affected.

### T-Cycles

As the circadian clock is not exactly 24 h, it is adjusted on a daily basis by the prevailing LD cycle. This entrainment process can be challenged using LD cycles whose length differs from 24 h – termed “T cycles”. A range of different T cycles have been used, where T refers to the day length. For example, an 11 h light, 11 h dark LD cycle is referred to as T22.

#### Circadian

The process of entrainment is limited and can only occur over a relatively narrow range—typically 23–25 h in mammals. Under short T cycles, the animal has to constantly accelerate its internal clock; whereas under long T cycles, the internal clock must be decelerated. As a result, the phasing of activity relative to the LD cycle may also change. Outside the range of entrainment animals will show a non-entrained period ~24 h despite the prevailing LD cycle. A second period corresponding to the period of the LD cycles may also occur. Rhythms in different aspects of physiology and behavior may correspond to the period of the LD cycle or the ~24 h period (212). T cycles have been used to study the negative consequences of circadian disruption on healthy physiology in humans, including effects on cognitive performance (97, 213). However, it should be noted that the T cycle studies performed in nocturnal rodents often differ from the classical forced desynchrony protocols used in human subjects. As animal studies often use higher light levels, animals are periodically exposed to relatively bright stimuli. This results in repeated phase shifting, and as the phase response curve of nocturnal rodents largely results in delays, this produces a non-entrained period which is slightly longer than the normal free-running period (214, 215).

#### Sleep

Studies on rats under 22 h days result in desynchrony with animals showing both 22 and >24 h rhythms in activity, sleep/wake, and NREM sleep. However, rhythms of core body temperature and REM sleep were desynchronized and predominantly cycled with a period >24 h (73). Studies using extreme T7 cycles report that total sleep levels and sleep distribution were unaffected (122). However, recent studies using spectral analysis of EEG signals from mice under 20- to 22 h T cycles show that these conditions result in higher SWA during sleep, as well as changes in the power of theta and gamma frequencies during waking (216).

#### Arousal

Plasma corticosterone has been reported to be rhythmic but elevated under T7 cycles (122).

#### Cognitive

22 h T cycles have been reported to impair passive avoidance memory but exert no effect on recognition memory (217). Other studies have found that 20 h T cycles impair reversal learning but result in no effects on Morris watermaze performance (8). Finally, T7 cycles have been reported to affect both watermaze and recognition memory (122).

As the above summary shows, T cycles can produce complex effects on physiology. As different aspects of physiology and behavior may devolve to the period of the LD cycle or the period of the circadian clock, this can give rise to internal desynchrony. Moreover, T cycles can result in a dynamically changing relationship between internal biological time and the external LD cycle. When out of phase, physiology and behavior may be dramatically affected, whereas when re-aligned they may appear relatively normal. As shown by recent data on sleep, the disruption of sleep/wake timing that results from housing under long-term T cycles can result in increased homeostatic sleep pressure and consequences for subsequent waking behavior (216). In conclusion, while T cycles provide valuable experimental tools for studying the relationships between biological clocks and the light environment, effects on sleep and arousal must also be considered when subsequently studying cognitive processes.

### Dim Light at Night

Due to the widespread adoption of artificial light, nocturnal light exposure is increasingly common. To study the physiological consequences of light exposure during the dark phase, protocols have been used in which animals are exposed to light during their normal dark (active) phase.

#### Circadian

Dim light at night has been shown to reduce locomotor activity levels, with no change in daytime activity (218, 219). Periodogram power was found to be reduced, and rhythms of PER1 and PER2 immunoreactivity in the SCN were found to be blunted (218).

#### Sleep

Data suggest that dim light at night does not affect sleep in mice, with no changes in either sleep timing or SWA (220). However, studies in rats report a decreased amplitude of daily rhythms of REM and NREM sleep as well as specific changes in the NREM EEG spectra around 16–19 Hz (221).

#### Arousal

Rhythms of corticosterone were found to be blunted under dim light at night conditions (218).

#### Cognitive

Initial studies of light at night often involved exposure to constant light (LL), but with the provision of an opaque tube to provide an escape to minimize any effects related to stress/arousal. Under such conditions, mice show increased anxiety (elevated plus maze and open field tests) and increased depression-like behaviors (forced swim test and sucrose anhedonia) (187). In hamsters, similar depression-like behaviors were also observed under light/dim light cycles, in which animals were housed under 150 lux during the day but 5 lux during the night (rather than complete darkness). By contrast, anxiety responses were reduced in hamsters housed under these conditions (222). The effects of dim light at night have been reported to be wavelength dependent, with blue-enriched dim light at night having greater effects than red-enriched light (219). Studies in a diurnal rodent, the Nile grass rat, showed similar depression-like effects of dim light at night, coupled with impaired learning and memory, assessed using the Barnes maze (223). Depression-like responses were increased in mice under dim light at night conditions, though the previously described effects on learning and memory were not detected (224).

In summary, dim light at night protocols provide another alternative approach to studying the effects of circadian disruption, producing effects on anxiety and depression-like behavior, with more subtle effects on sleep. However, the effects of these protocols on cognitive processes are more limited, and effects on learning and memory are not observed in mice, suggesting species differences may exist. Differences in the physiological and behavioral effects of dim light at night compared to other circadian disruption protocols may provide insight into the different pathways by which aberrant lighting influences physiology and behavior. For example, the effects of dim light at night on anxiety and depression-like behaviors with blunted corticosterone rhythms appear qualitatively different from the effects on learning and memory with disrupted sleep and elevated arousal that accompany jet-lag and T cycle conditions.

### Disruptive Phase Shift

A final protocol that has been used to study circadian disruption in Siberian hamsters (*Phodopus sungorus*) is the use of DPSs.

#### Circadian

Hamsters show phase shifting responses similar to other nocturnal rodents, with light exposure during the early subjective night giving rise to phase delays in activity and light exposure during the late subjective night producing phase advances. However, when Siberian hamsters were housed under 16:8 LD cycles then exposed to a combination of two 15-min light pulses, the first advancing and a second delaying light pulse the following day, this led to a compression of activity and long-term arrhythmicity in the majority of animals. Rhythms in activity, body temperature, and melatonin were all affected, and hamsters remained arrhythmic even when subsequently exposed to normal LD cycles (225). Studies using a 2 h advancing light pulse followed by a 3 h phase delay in the LD cycle produced similar irreversible arrhythmia within a few days, and these effects were associated with reductions in clock gene expression in the SCN (226).

#### Sleep

Sleep has been studied in DPS hamsters to investigate sleep homeostasis in the absence of circadian input. In arrhythmic hamsters, the usual difference between sleep during the light and dark was no longer apparent, with high levels of daytime sleep. Arrhythmic animals also show an increase of around 1.5 h of sleep per day compared with rhythmic controls. No differences in sleep homeostasis were detected (227).

#### Arousal

While we were unable to find any studies on adrenal glucocorticoids under these conditions, given the effects of DPS protocols on other aspects of circadian and neuroendocrine function, it is expected that normal rhythmic glucocorticoid rhythms will be abolished.

#### Cognitive

Novel object recognition in hamsters shows a circadian rhythm, with increased performance during the subjective night. However, these rhythms were found to be impaired in arrhythmic hamsters (228). Subsequent studies showed that DPS-induced arrhythmicity resulted in impaired novel object recognition and spontaneous alternation. By contrast, SCN lesioned animals showed no impairments in these tasks. Surprisingly, if DPS-treated hamsters were SCN lesioned, their impaired performance was reversed, despite the animals remaining arrhythmic. These findings suggest that the SCN may influence memory *via* inhibitory output, which impairs performance when SCN function is compromised (229).

Disruptive phase shift protocols have provided a valuable contribution to the understanding of the role of circadian rhythms in the regulation of learning and memory. Although these effects are species specific, they provide an important alternative arrhythmic model to SCN lesioning or clock gene knockouts that are commonly studied in mice. Due to its profound effects, the DPS hamster model provides a better model of arrhythmia rather than circadian disruption due to aberrant light exposure.

## Future Perspectives

Advances in our understanding of the photoreceptors mediating the effects of light on physiology and behavior have provided a greater appreciation of how different systems may interact to regulate complex downstream processes, such as cognition. However, this highlights the need for multiple physiological systems, including circadian rhythms, sleep, and arousal, all to be considered when interpreting the effects of any intervention on such complex behavioral outcomes. Despite the number of studies of circadian rhythms in learning and memory, the effects of preceding sleep at different circadian times is typically not considered. In turn, when sleep is studied, circadian processes are often overlooked. Coupled to these issues, arousal state is rarely considered, and this can have profound effects on cognitive outcomes—resulting in either improved or impaired performance—and making findings difficult to interpret. As a result, our understanding of the mechanisms by which light modulates cognitive processes, and how abnormal light exposure disrupts these processes, is often limited.

The development of methods for simultaneously monitoring circadian activity and sleep in the home cage provides one approach to account for circadian rhythms and sleep throughout behavioral studies (230). While this allows the timing and duration of sleep to be assessed over multiple cycles, it does not, however, provide detailed spectral information. Inclusion of markers such as adrenal glucocorticoids provides one way of assessing arousal, though measurements of other parameters such as markers of cardiovascular function also be informative. Markers of generalized arousal, such as spontaneous fluctuations in locomotor activity may also be of value in this regard (169, 170). While improved biomarkers of arousal are certainly needed, it should also be considered that arousal may not be a unitary process. More work in this area is clearly required.

While a range of different protocols have been used to produce experimental circadian disruption in rodents, these cannot be assumed to be directly comparable. Even where the same protocols are used, these may differ in the light levels, spectral composition and photoperiods used to induce circadian disruption. Some protocols are only effective in specific species (or even strains), making generalization difficult. Coupled to these issues, different behavioral tests have been used to measure learning and memory as well as other behavioral responses to circadian disruption, which may involve different cognitive processes.

Another major issue facing the field is adopting standard metrics to define sleep and circadian rhythm disruption. Circadian biologists have traditionally focused on the measurement of circadian period, whereas sleep researchers analyze the frequency and power of EEG spectra. Both fields have developed refined analytical tools to measure these processes of interest. However, a common feature of circadian disruption—particularly that observed in disease—is the fragmentation of normal physiological and behavioral rhythms. In the circadian field, light phase activity, phase angle of entrainment and periodogram amplitude have been variously used as markers of circadian disruption. In both sleep and circadian research, the number or duration of bouts is also measured. Other measures of disruption include inter-daily stability and intra-daily variability, which assess day-to-day reproducibility of rhythms and the frequency of transitions between rest and activity, respectively (231). Standard approaches to the measurement of circadian disruption are clearly required. The increasing requirement of journals and funders to deposit raw data may facilitate the development of new analytical tools in this area, as well as better enabling comparison between studies.

**Figure 4:**
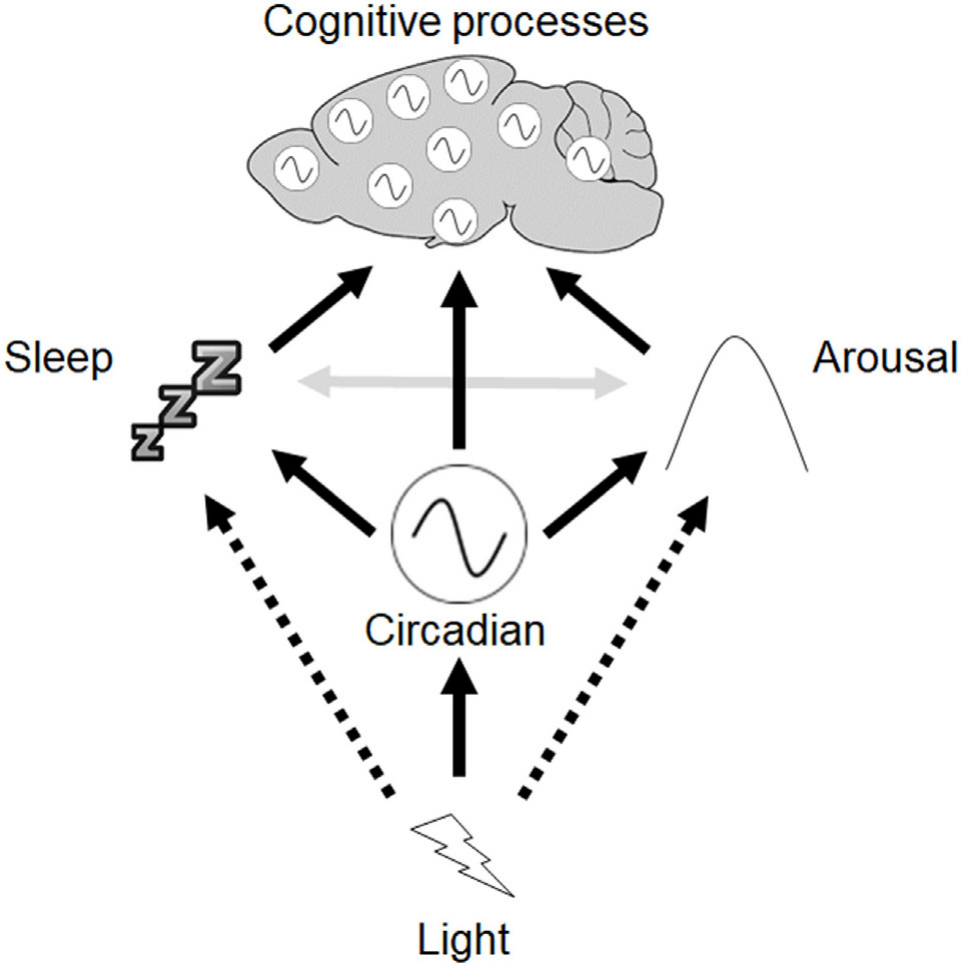
Framework to describe the interactions between circadian rhythms, sleep, and cognition. Light exerts direct effects on the circadian clock in the suprachiasmatic nuclei (SCN), which in turn modulates other rhythmic processes throughout the body, including independent oscillators found in other brain regions. The SCN clock also modulates sleep, and regulates arousal via output to hypothalamic-pituitary-adrenal axis and sympathetic nervous system. Light may also directly regulate sleep as well as arousal (dashed arrows). Reciprocal interactions between sleep and arousal also occur (gray arrows).

While the overall consensus is that circadian disruption resulting from different LD protocols can give rise to cognitive impairment, the results depend upon the specific protocol used. We propose a framework to help conceptualize how light may influence circadian rhythms, sleep, and arousal to modulate cognitive processes (Figure 4). Light exerts direct effects on the circadian clock in the SCN, which in turn modulates other rhythmic processes throughout the body, including independent oscillators found in multiple different brain regions. The SCN clock also modulates sleep, and regulates arousal *via* output to the HPA axis and sympathetic nervous system. Light may also directly regulate sleep as well as arousal. Sleep and arousal are also reciprocally linked. Finally, acute and chronic changes in lighting conditions may exert different effects.

It is unlikely that any single mechanism mediates the effects of light on cognitive processes, and instead light may exert its effects on a network of interacting processes. This includes the direct effects of light on physiology and behavior as well as the modulation of circadian rhythms. Despite the widespread use of different abnormal light exposure protocols to study the effects of circadian disruption on cognition, such protocols have very different effects on circadian rhythms, sleep, and arousal, with subtly different consequences for different aspects of cognition. As a result, we propose that any studies addressing the effects of light on different cognitive processes should account for their effects on circadian rhythms, sleep, and arousal if we are to properly understand the physiological basis of these effects.

## Author Contributions

AF and SP prepared the manuscript, with edits based on feedback from ST, LB, VV, and DB.

## Conflict of Interest Statement

The authors declare that the research was conducted in the absence of any commercial or financial relationships that could be construed as a potential conflict of interest.
